# Supplementary material for: The impact of racially-targeted food marketing and attentional biases on consumption in Black adolescent females with and without obesity: Pilot data from the Black Adolescent & Entertainment (BAE) study
Source: PLoS One. 2023 Jan 20;18(1):e0279871. doi: 10.1371/journal.pone.0279871 (PMC9858861; doi:10.1371/journal.pone.0279871)
Supplement: Attachment — Submitted filename: RT food marketing and AB_PlosOne_RR2_11.11.2022_coverletter.docx [file pone.0279871.s002.docx]

November 10, 2022

Ali B. Mahmoud, Ph.D.
Academic Editor
PLOS ONE

Dear Dr. Mahmoud,

We appreciate another opportunity to revise and resubmit our manuscript entitled, “**The impact of racially-targeted food marketing and attentional biases on consumption in Black adolescent females with and without obesity: Pilot data from the Black Adolescent & Entertainment (BAE) Study”** (PONE-D-21-07262R1).

We have outlined our responses to Reviewer #3’s comments. We have placed the comments in **bold** and our responses in normal font:

Review Comments to the Author

**Reviewer #3: Please note: I am reading this paper for the first time after its first round of revisions. Apologies if any of my recommendations contradict previous reviewers' - which can happen!**

**Small note - Unhealthy food advertisements (“ads”) suggested to me that “ads” is short for the 3-word phrase; suggest rewriting**

We have added, ‘ “advertisements” hereafter referred to as “ads” ’ to clarify the shortened phrase.

**Abstract - I found some aspects of the Abstract confusing and suggest it could be clarified by attending to sequencing of information

1. ‘Fifty-five (50%) adolescents qualified for the study.’ What enabled them to qualify and what were their characteristics vs those who did not? Is this information key to the study outcomes? The presence of 'qualified' suggests it is. Earlier it states participants were N=31; later that 87% participated; and then that all eligible participants completed the visit. I can’t see how these figures are related to one another. Having read on I see that the method states N=41, so perhaps above is a typo, and this explains 87%; but the other questions remain. Furthermore, some of the above information is not given in the Method, which doesn’t help. I suggest you remove most of this from the Abstract, giving only the correct participant number, and saving Abstract word count for more substantive matters. And explain about recruitment and participation etc in the Method.**

We have edited the sample size typo and removed most of these details from the Abstract, which now states (lines 53-73):

Unhealthy food advertisements (“advertisements” hereafter referred to as “ads”) are linked to poor diet and obesity, and food companies disproportionally target Black youth. Little is known about the mechanisms whereby food ads influence diet. One possibility may be racially-targeted ads that appeal to Black youth. Those with food-related attentional biases may be especially vulnerable. The objective of this project was to assess the feasibility and initial effects of a pilot study testing the influence of racially-targeted food ads and food-related attentional biases on eating behaviors among a sample of Black adolescent females. Feasibility of recruitment, retention, and procedures were examined. Participants (*N*=41, 12-17y) were randomized to view a television episode clip of the *Big Bang Theory* embedded with either four 30-second racially-targeted food ads or neutral ads. A computer dot probe task assessed food-related attentional biases. The primary outcome was caloric consumption from a laboratory test meal. Interactions based on weight and ethnic identity were also examined. Analyses of variance and regressions were used to assess main and interaction effects. Exposure to racially-targeted food ads (versus neutral ads) did not affect energy consumption (*p* > .99). Although not statistically significant, adolescents with obesity consumed nearly 240 kcal more than non-overweight adolescents (*p* = 0.10). There were no significant preliminary effects related to food-related attentional biases or ethnic identity (*p*s=0.22-0.79). Despite a non-significant interaction, these data provide preliminary support that adolescents with obesity may be particularly vulnerable to racially-targeted foods ads. An adequately powered trial is necessary to further elucidate the associations among racially-targeted food ads among Black adolescent girls with obesity.

Information about the recruitment is included in the Results (versus the Method) per the suggestion of a previous reviewer. The paragraph states (lines 294-303):

We recruited a total of 167 adolescents primarily through direct mailings and pre-screened 89% of the potential participants (Fig 1). After excluding 56 (50.4%) of the potential participants, the remaining 55 (49.5%) adolescents were scheduled for a visit. Most (*n* = 48, 87.3%) attended the visit and signed consents/assents. The research team scheduled three touch points between the initial contact and study visit. These interactions provided continuous engagement with potential participants and the opportunity to reschedule visits, if needed. One hundred percent of adolescents reported following instructions to fast and not exercise two hours before the visit and 100% of eligible participants completed the entire visit. Although all participants completed the AB task, seven (22.6%) participants were excluded from analyses due to high error rates.

**2. ‘Despite a non-significant interaction, preliminary data suggested that adolescents with
obesity may be particularly vulnerable to racially-targeted foods ads (p=0.17)’ Again, step-wise sequencing of information would clarify. Suggest you lead with main effects and then interaction, or non-significant findings and then significant ones – at present it is difficult to absorb the findings quickly, which is what one needs from an Abstract.**

We have updated the Abstract to provide a step-sequencing of information about the results (lines 64-69):

Analyses of variance and regressions were used to assess main and interaction effects. Exposure to racially-targeted food ads (versus neutral ads) did not affect energy consumption (*p* > .99). Although not statistically significant, adolescents with obesity consumed nearly 240 kcal more than non-overweight adolescents (*p* = 0.10). There were no significant preliminary effects related to food-related attentional biases or ethnic identity (*p*s=0.22-0.79).

**3. Finally, the concluding sentence of the Abstract is rather general. Something that indicates which data and which direction this is taking you would help the reader.**

We have edited the concluding sentence to the following (lines 69-73):

Despite a non-significant interaction, these data provide preliminary support that adolescents with obesity may be particularly vulnerable to racially-targeted foods ads. An adequately powered trial is necessary to further elucidate the associations among racially-targeted food ads among Black adolescent girls with obesity.

**4. Participant characteristics –Was this itself an outcome? Or was the hypothesis about recruitment, retention, completion? Either way it would make more sense to section out the Results according to hypotheses/research questions so the purpose of each section is clear.**

The participant characteristics section provides the relevant demographic data, but was not itself an outcome. The Results have been re-organized based on aims to make each section and the analyses clearer. See lines 281-353.

**5. ‘285 Based on parents’ occupations, the median income was $96,110’ etc What is the source for this? Furthermore, it’s difficult to gain a rapid overview of this information in the narrative format. I would consider placing all the relevant demographic information in a single table.**

The source of the estimated income is from the 2018 national wage estimates provided by the U.S. Bureau of Labor Statistics (citation added; lines 214-215). Upon further reflection, we recognized that percentage breakdown is not relevant for these analyses and so has been removed.

**6.‘304 …. One hundred percent of adolescents followed instructions to fast and 305 not exercise before the visit’ I assume it might be more accurate to say that they reported having followed the instructions?**

The sentence now states (lines 299-301), “One hundred percent of adolescents reported following instructions to fast and not exercise…”

**7. ‘319... Average pre-visit self-reported intake was 363.40 kcal (SD = 258.40 kcal).’ How does this align with instructions to fast above?**

We asked participants not to fast two hours before their visit, so some participants may have eaten prior to the 2-hour window. This detail has been added for clarity (lines 299-301): “One hundred percent of adolescents reported following instructions to fast and not exercise two hours before the visit…”

**8. 336 onwards – this seems to repeat the findings just reported? Overall this section is really not very clear, and I had to read it several times to grasp it. I suggest you consider a rewrite with clear subheads and topic sentence introducing purpose of each analysis.**

We have reorganized these sections and created subheadings for clarity. See lines See lines 292-353.

**9. 371 – Big Bang Theory – perhaps say a popular show among adolescents at the time but one with an all-White cast? Among the young people I know, BBT has become quite discredited not just for its lack of diversity but also for its stereotyping of gender and neurodiversity.**

We now state (lines 368-371):

For instance, youth viewed an episode of the *Big Bang Theory*, a popular show among adolescents at the time of the study that included a predominantly White cast. Other TV shows that resonate with Black participants will likely be more suitable for the larger study.

**9. Discussion – I think you could consider discussing the literature on media effect sizes and issues about how sub-group effects are washed out in all-group analyses. Overall, a stronger account of the meaning and implications of this complex study, linking it to current literature and pointing forward to future work, would strengthen the paper in my view.**

We have added a more thorough discussion of effect sizes (lines 376-394):

There were small-to-medium effect sizes observed, and given the small sample size, it was difficult to reach significance. While efforts are typically made in psychological research to find ways to increase effect size to improve probability of finding an effect (if one exists), smaller effect sizes may still have practical significance. It is common in biomedical clinical trials investigating serious diseases (e.g., heart attacks) to conclude that certain treatments are effective, even if the effect sizes are small [55]. The perspective of these researchers is that the benefit of even a small reduction in the risk of a heart attack significantly outweighs the risk of not being treated. While no study to date has examined food marketing research using this lens, it may be helpful to take a similar perspective. Even a small increase in the amount of food consumed after being exposed to a food ad may underscore a risk that may outweigh the benefit of not making any changes to the current food environment. Even with a small effect size, if risks outweigh benefits, it is important for changes to be considered. It is can also be argued that a small change detected during one meal, compounded across all meals over the course of an entire lifetime is substantial. If such an impact is again compounded across millions of people, the economic impact may be quite large. More research is needed to quantity the population impact of food marketing research with smaller effect sizes. Black adolescents remain key targets of food and beverage marketing, despite nationwide efforts to address obesity and eating issues [1]. The current findings can provide information that can support future research in this area.

We added a comment about the limitations of sub-group analyses to the Limitations (lines 408-409), “Further, with small sample sizes, subgroup analyses can be misleading, and thus we have interpreted these data with caution.”

Given the small sample size and focus on preliminary examinations, prior reviewers cautioned against making any additional interpretations and implications from these data.

**10. Some Minor things: 83 ‘or feature Black actors’ should read ‘or featuring’ 115 ‘towards fwdood’**

Corrected.

We appreciate the Reviewer’s comments and hope our updates have strengthened the manuscript.

Best,

Omni Cassidy, Ph.D.
